# Modulation of Host Immune Response during *Leishmania infantum* Natural Infection: A Whole-Transcriptome Analysis of the Popliteal Lymph Nodes in Dogs

**DOI:** 10.3389/fimmu.2021.794627

**Published:** 2022-01-04

**Authors:** Carolina R. Sanz, Guadalupe Miró, Natalia Sevane, Armando Reyes-Palomares, Susana Dunner

**Affiliations:** ^1^ Animal Health Department, Veterinary Faculty, Complutense University of Madrid, Madrid, Spain; ^2^ Department of Animal Production, Veterinary Faculty, Complutense University of Madrid, Madrid, Spain; ^3^ Department of Biochemistry and Molecular Biology, Complutense University of Madrid, Madrid, Spain

**Keywords:** canine leishmaniosis, immunomodulation, parasite immune evasion, unfolded protein response, cytokine, T cell exhaustion, RNA-seq, lncRNA

## Abstract

*Leishmania infantum*, the etiological agent of canine leishmaniosis (CanL) in Europe, was responsible of the largest outbreak of human leishmaniosis in Spain. The parasite infects and survives within myeloid lineage cells, causing a potentially fatal disease if left untreated. The only treatment option relies on chemotherapy, although immunotherapy strategies are being considered as novel approaches to prevent progression of the disease. To this aim, a deeper characterization of the molecular mechanisms behind the immunopathogenesis of leishmaniosis is necessary. Thus, we evaluated, for the first time, the host immune response during *L. infantum* infection through transcriptome sequencing of the popliteal lymph nodes aspirates of dogs with CanL. Differential expression and weighted gene co-expression network analyses were performed, resulting in the identification of 5,461 differentially expressed genes (DEGs) and four key modules in sick dogs, compared to controls. As expected, defense response was the highest enriched biological process in the DEGs, with six genes related to immune response against pathogens (*CHI3L1, SLPI, ACOD1, CCL5, MPO, BPI*) included among the ten most expressed genes; and two of the key co-expression modules were associated with regulation of immune response, which also positively correlated with clinical stage and blood monocyte concentration. In particular, sick dogs displayed significant changes in the expression of Th1, Th2, Th17 and Tr1 cytokines (e. g. TNF-α, IFN-γ, IL-21, IL-17, IL-15), markers of T cell and NK cell exhaustion (e. g. *LAG3, CD244, Blimp-1, JUN*), and B cell, monocyte and macrophage disrupted functionality (e. g. *CD40LG, MAPK4, IL-1R, NLRP3, BCMA*). In addition, we found an overexpression of *XBP1* and some other genes involved in endoplasmic reticulum stress and the IRE1 branch of the unfolded protein response, as well as one co-expression module associated with these processes, which could be induced by *L. infantum* to prevent host cell apoptosis and modulate inflammation-induced lymphangiogenesis at lymph nodes. Moreover, 21 lncRNAs were differentially expressed in sick dogs, and one key co-expression module was associated with chromatin organization, suggesting that epigenetic mechanisms could also contribute to dampening host immune response during natural *L. infantum* infection in the lymph nodes of dogs suffering from clinical leishmaniosis.

## Introduction

Leishmaniosis is a zoonotic disease caused by *Leishmania* spp. and transmitted by blood-sucking phlebotomine sand flies. Despite affecting 200,000–400,000 people annually and causing an estimated 20,000–40,000 deaths per year, leishmaniosis is still one of the most neglected diseases in the world (1–3). *L. infantum* was identified as the causative agent of the largest outbreak of human leishmaniosis in Spain (4), which is also the aetiological agent of canine leishmaniosis (CanL) in Europe. This protozoan is an obligate intracellular parasite that lives within myeloid lineage cells. It is capable of reprogramming the host microenvironment to invade and persist within the mammalian host cells, causing a systemic, chronic, and potentially fatal disease if left untreated (5–8).

Treatment of this zoonotic disease is a major challenge as the only option relies on chemotherapy (9, 10), and the emerging anti-leishmanial drug resistances, coupled with long duration of treatments and drug toxicity, further limit its efficacy (11). Immunotherapy, in conjunction with anti-leishmanial drugs (immunochemotherapy), could result in a synergic effect with activation of the protective immunity of the host and direct action of drugs against the parasite. Thus, immunotherapy and/or immunochemotherapy might be a promising alternative approach for treating leishmaniosis (12–15). In this regard, the use of vaccines as immunomodulatory agents could also help to reduce the parasite burden in infected dogs (13). However, a deeper characterization and a better understanding of the complex molecular mechanisms behind the immunopathogenesis of *L. infantum* infection are necessary to successfully develop efficient immunomodulatory drugs and treatment strategies.

Currently, it is widely accepted that a delicate balance between inflammatory and regulatory responses is required to achieve the immune control of *L. infantum* (12). Specifically, control of the infection relies on a successful macrophage activation via interferon-γ (IFN-γ), produced mainly by proinflammatory CD4+ T helper type 1 (Th1) cells and natural killer (NK) cells stimulated by interleukin (IL)-12, that promotes leishmanicidal activity mediated by nitric oxide (NO) (12, 15–17).

In contrast, the parasite’s survival is associated with a predominant immunosuppressive response mediated by CD4+ T regulatory type 1 (Tr1) cells and populations of regulatory B cells. These cells produce IL-10 and transforming growth factor-β (TGF-β) (18), decreasing the proliferation of Th1 cells producing IFN-γ, and then resulting in a lack of M1 macrophage activation and parasite killing (12, 19, 20), which might be also correlated with an increase on the alternatively activated macrophages (M2). M2 macrophage polarization will result in the induction of IL-10 and the inhibition of proinflammatory signals (5). Additionally, it has been shown that an incomplete activation or exhaustion of CD8+ cytotoxic T lymphocytes (CTLs) and NK cells could also limit IFN-γ production and contribute to a more severe immunological imbalance, favoring the parasite persistence (21).

Other cells, such as neutrophils, are additional components involved in the immune response during *Leishmania* infection. Although they are the first cells recruited to the infection site, phagocytosis of *Leishmania* amastigotes by neutrophils could lead to both parasite elimination, *via* neutrophil oxidative burst, or to prolonged parasite survival within the parasitophorous vacuole, avoiding inflammatory signals (12, 22, 23). Furthermore, neutrophils release networks of extracellular fibers composed of chromatin and granular proteins, also known as neutrophil extracellular traps (NETs) (24), that may help some *Leishmania* species to escape from extracellular effector immune mechanisms and to stimulate M2 macrophages.

Even though these host-parasite interactions during *Leishmania* infection have been well-studied in various species, research and knowledge gaps remain, mainly regarding the precise mechanisms involved in the immune evasion of the parasite. Recent advances in next-generation sequencing, specifically transcriptomics, help to overcome the drawbacks of traditional methods and allow to expand the knowledge about the immunopathogenesis of diseases (25–28). Furthermore, emerging evidence suggests that intracellular pathogens can modulate or even hijack their host gene expression processes through non-coding RNA-mediated regulatory mechanisms as an additional strategy to dampen the host immune response (29, 30). Non-coding RNAs (ncRNAs), which consist of microRNAs (miRNAs) and long ncRNAs (lncRNAs), are transcripts that do not encode proteins but still act as global and crucial biological regulators (29–31). In particular, the expression of lncRNAs, defined as transcripts longer than 200 nucleotides with no protein-coding potential, are either up- or down-regulated during infections, enhancing the host immunity, or even promoting pathogen invasion or replication within the host cells (29). Thus, they could be involved in the immune evasion during *L. infantum* infection.

Here, we performed transcriptome analyses for deep profiling of molecular basis of the host immune response during *L. infantum* infection in the dog. Our main goal was to evaluate gene expression signatures in the popliteal lymph nodes of dogs with CanL compared to controls. Our data showed that *L. infantum* infection induces strong transcriptional changes in this tissue, which could regulate host immunity at multiple levels to promote parasite persistence. These results provide new insights into the underlying mechanisms behind CanL and pinpoint potential targets for novel therapeutic strategies.

## Materials and Methods

### Study Design and Sample Collection

We recruited client-owned dogs with a diagnosis of CanL of any age, breed, or gender attending to the Consultant of Infectious diseases at the Complutense Veterinary Teaching Hospital of Madrid, Spain. Informed consent was obtained from each dog’s owner before enrollment in the study. The main inclusion criteria were a positive serology result for *L. infantum* by immunofluorescence antibody assay (IFA) plus a positive cytology and a PCR result obtained from bone marrow or lymph node aspirates, as well as presenting with clinical signs or clinicopathological abnormalities associated with clinical stages II-III, according to LeishVet guidelines (32). Animals were excluded if they met any of the following conditions: (1) current or recent (past 90 days) treatment for CanL (e.g., allopurinol, methylglucamine antimoniate, miltefosine) or immunomodulators, such as domperidone, ciclosporin and/or corticosteroids; (2) current or recent (past 90 days) use of any kind of special diet or supplements to improve their immune response; (3) vaccinated against CanL; (4) current or recent (past 90 days) use of any drugs except flea’s, heartworm, and/or tick prevention; (5) evidence of secondary immune-mediated disease (e.g., neoplasia, other infectious diseases) based upon imaging studies or infectious disease serology/PCR testing; (6) under one year of age; (7) pregnant or lactating females. For each case, we recorded breed, age, sex, clinical signs, relevant laboratory values and clinical stage, based on LeishVet guidelines (32).

Healthy control dogs were also recruited considering the following inclusion criteria: (1) negative serology result for *L. infantum* by IFA test; (2) negative cytology and/or PCR result obtained from bone marrow or lymph node aspirates; (3) an unremarkable physical examination performed by a veterinarian; (4) absence of clinicopathological abnormalities. Exclusion criteria for control dogs were the same as for infected/sick dogs. For each control dog, we recorded breed, age, and sex.

**Figure 1:**
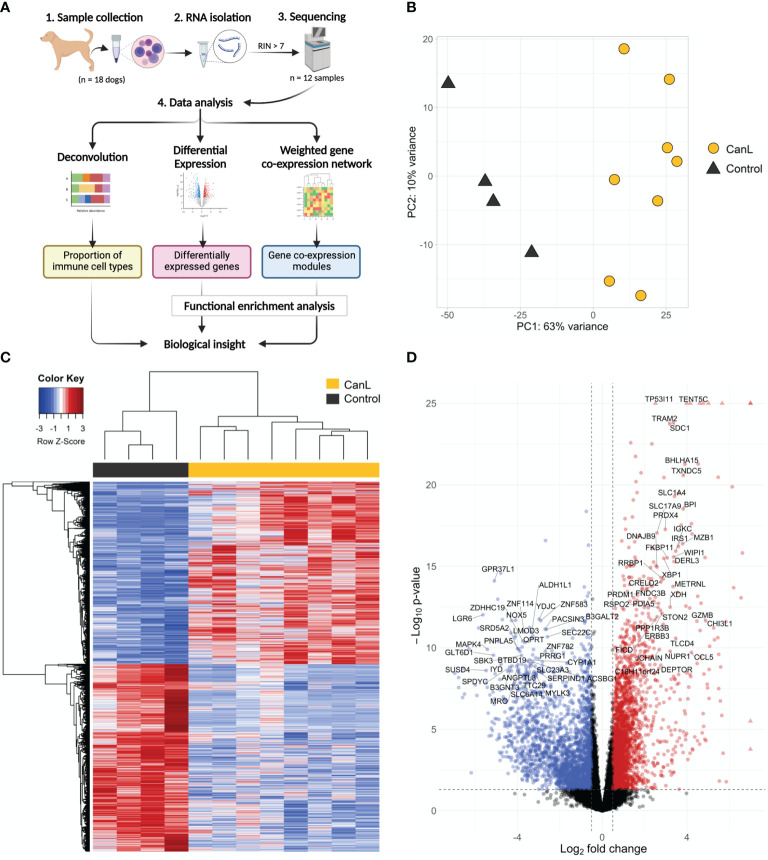
**(A)** Overview of the study workflow (created with BioRender.com). **(B)** Principal component analysis of the samples showing PC1 and PC2. **(C)** Heatmap of the most variable genes expressed in dogs with CanL vs. healthy dogs. Expression profiles for the 1,500 genes with the highest variability that shown significant expression changes in dogs with CanL (right) and healthy dogs (left). Red represents genes overrepresented in CanL samples, and blue indicates genes overrepresented in controls. **(D)** Volcano plot where mean log_2_FC is plotted against the –log_10_ FDR adjusted P-values for all expressed genes. Each point represents a gene, and those with FDR < 1x10^-26^ and/or log_2_FC > 7 are displayed as triangles. Genes that reach the cut-off values (FDR < 0.05 and absolute log_2_FC > 0.5) are highlighted. Labels are displayed for the most significant (FDR < 10^-8^ and absolute log_2_FC > 2.5) protein-coding genes.

In order to perform aspirates from popliteal lymph nodes, hairs were removed from the skin over the site of puncture and asepsis was done with an alcoholic solution of 2% chlorhexidine. The lymph node aspirates were done using a 25 x 20 mm needle and a 10 ml syringe (**Figure 1A**). The lymph node samples (approximately 50 µl) were preserved in 200 µl of RNAlater (Qiagen, Valencia, CA) and stored according to the manufacturer’s guidelines. For infected/sick dogs, lymph node aspirates were collected prior to any therapeutic intervention.

### Total RNA Isolation, Library Preparation, and Sequencing

Total RNA was extracted from the lymph node samples with the RNeasy mini kit (Qiagen) with on-column DNase I treatment according to the manufacturer’s protocol (**Figure 1A**). Concentration and integrity of extracted RNA were measured with a NanoDrop ND-1000 Spectrophotometer (NanoDrop Technologies, Wilmington, DE) and an Agilent 2100 Expert Bioanalyzer (Agilent, Santa Clara, CA), respectively. Samples with an RNA Integrity Number (RIN) < 7 were excluded from downstream analyses. Approximately 1 μg of RNA from each dog was submitted for library preparation and sequencing at the DNA Link Sequencing Lab (Republic of Korea). cDNA libraries were constructed using the TruSeq Stranded Total RNA library Preparation Kit (Illumina, San Diego, CA), and a minimum of 5 Gb RNA-seq data per sample were generated using a NovaSeq 6000 System (Illumina) in paired-end read, 100 bp run mode. Base calling was done by Real Time Analysis (Illumina), and the output was demultiplexed and converted to FASTQ format with Bcl2fastq (version 2.20; Illumina).

### RNA-Seq Data Processing

Raw paired-end reads were checked for a possible low base score, Illumina sequencing adapters and PCR contaminations with FastQC v0.11.9 (33). Illumina sequencing adapters and low quality sequences were removed with Trimmomatic v0.39 (34).

Illumina-RNA sequencing reads were pseudo-aligned to the Ensembl 98 CanFam 3.1 reference transcriptome (35) using Salmon v1.1.0 (36). Expression per gene were summarized using tximport v1.14.2 (37) in R v4.1.0 (38) and Bioconductor v3.13 (39).

Genes with a mean raw count lower than 5 across all samples were removed for downstream analyses. Raw gene counts were normalized and variance stabilized for exploratory data analysis using principal component analysis (PCA) of the 1,500 most variable genes.

### Deconvolution of RNA-Seq Data

To estimate the abundance of immune cell subtypes and account for potential tissue heterogeneity (**Figure 1A**), we used the human validated signature matrix LM22 to deconvolute the bulk lymph node gene expression mixture matrix of orthologous human genes, in Fragments Per Kilobase of exon model per Million reads, with CIBERSORTx online tool (40). The LM22 matrix contains a total of 547 genes for distinguishing 22 hematopoietic cell subsets, including seven T cell types, naïve and memory B cells, plasma cells, NK cells and myeloid subsets. It was generated using Affymetrix HGU133A microarray data (41), but it has been rigorously tested for the application to RNA-Seq data and immune monitoring when samples cannot be immediately processed (40–42), even for non-human specimens (43–45). The fractions of immune cell types were compared across different groups using Wilcoxon test, and considering a statistical significance threshold of p-value ≤ 0.05.

### Differential Expression Analysis

Differential expression analysis was performed using DESeq2 v1.26.0 (46) between uninfected versus infected samples (**Figure 1A**), considering as a significant expression change when the False Discovery Rate (FDR) ≤ 0.05. Additional gene annotation was obtained using biomaRt v2.42.1 (47) to access canine reference transcriptome (CanFam 3.1), such as biotype (e.g. protein-coding or non-coding RNA) and gene names.

### Weighted Gene Co-expression Network Analysis

Gene co-expression analysis was performed to identified highly co-regulated modules across infected samples (clinical stages II-III) using WGCNA package v1.46 (48) (**Figure 1A**). Briefly, we built a gene co-expression network of genes with a mean of normalized counts across samples ≥ 7 by calculating Pearson’s correlations between their expression values. Subsequently, a weighted adjacency matrix was established by raising the co-expression similarity to apply a soft threshold power (β) of 12 to fit a scale-free network model (49) (**Figure S1**). The topological overlap measure (TOM) and its corresponding dissimilarity (1-TOM) were calculated using the adjacency matrix, and (1-TOM) was used as a distance between genes for hierarchical clustering analysis.

Modules were defined using DynamicTreeCut algorithm (48) with the following parameters: deep split = 2, cut height = 0.25, minimal module size = 30 genes. Therefore, modules can be defined as clusters of highly interconnected genes –with high topological overlap– and each of them will be identified with a color. The module-trait relationships were also estimated by calculating the correlations between Module Eigengenes (ME), a summarized expression profile of the module and inter-connected with intra-modular genes, and clinical features of patients (e.g. clinical stage, age, sex, haematological and biochemical parameters) (**Table S1**). In addition, Pearson’s correlation between expression profile of each gene and ME was calculated to identify the Module Membership (MM). Modules were potential biologically interesting and significantly associated with the variables when they had a correlation > 0.6 and a p-value ≤ 0.01 with clinical traits.

Gene Significance (GS), defined as the absolute correlation between the gene and the trait, was used to quantify associations of individual genes with clinical traits. The Module Significance (MS), defined as the average absolute GS of all the genes involved in the module, was calculated to evaluate the association strength.

### Functional Enrichment Analysis

Functional enrichment analyses for biological processes in the Gene Ontology (GO) were performed either for differentially expressed genes and genes within modules using enrichGO and gseGO functions of clusterProfiler v4.1 (50) (**Figure 1A**). We used all expressed genes as background, and only annotated genes were included in the analyses. Later, multiple testing correction was applied using Benjamini-Hochberg method and selecting only as significantly enriched those GO terms with an adjusted p-value ≤ 0.05. The most significant GO categories of biological process were used to characterize the key modules.

## Results

### Clinical Features of the Patients

A total of 18 dogs met the inclusion criteria and were recruited for this study. Ten of these dogs were affected with CanL, and the other were healthy non-infected dogs which served as controls. The median age of animals included was 5 years, ranging from 1 to 12 years. The most represented dog breed was mixed breed, followed by American Staffordshire Terrier. Seven patients were clinically classified as stage II (moderate disease), and only three cases were in clinical stage III (severe disease). Complete clinical data is shown in (**Table 1**). Seven of the infected/sick dogs had been treated with leishmanicidal and/or leishmaniostatic drugs at some point before they were included in the study. However, none of them had received any treatment for CanL within the last 90 days prior to inclusion.

**Table 1:** Clinical data for enrolled cases and controls.

Group	Dog ID	Age (years)	Breed	Sex	IFA	PCR	Creatinine (mg/dL)	UPC	Clinical Stage	RIN	RNA-seq
Case	C7915	12	Mongrel	M	1/1600	Positive	0.90	3.19	III	9.0	Yes
	C7916	6	English Setter	M	1/1600	Positive	0.80	0.40	II	9.4	Yes
	C7917	5	Spanish Greyhound	M	1/800	Positive	0.60	0.10	II	6.3	No
	C7918	5	American Staffordshire Terrier	F	1/6400	Positive	1.00	0.40	II	9.2	Yes
	C7920	1	American Staffordshire Terrier	F	1/800	Positive	0.50	0.30	II	6.7	No
	C7921	9	Labrador Retriever	F	1/400	Positive	1.00	0.60	II	8.9	Yes
	C7975	7	Mongrel	M	1/1600	Positive	0.90	0.20	II	8.3	Yes
	C7977	2	Mongrel	M	1/6400	Positive	1.57	0.48	III	8.4	Yes
	C7978	5	Pug	F	1/6400	Positive	0.50	0.33	II	9.1	Yes
	C7982	6	Spanish Greyhound	M	1/800	Positive	0.90	2.93	III	8.4	Yes
Control	C8065	1	Bullmastiff	F	Negative	Negative	–	–	Non-infected	6.2	No
	C8066	4	Labrador Retriever	M	Negative	Negative	–	–	Non-infected	7.8	Yes
	C8069	5	Labrador Retriever	F	Negative	Negative	–	–	Non-infected	6.4	No
	C8071	2	Labrador Retriever	F	Negative	Negative	–	–	Non-infected	7.7	Yes
	C8072	12	Yorkshire Terrier	F	Negative	Negative	–	–	Non-infected	7.0	Yes
	C8073	6	Dalmatian	M	Negative	Negative	–	–	Non-infected	8.5	Yes
	C8075	8	Mongrel	F	Negative	Negative	–	–	Non-infected	4.8	No
	C8208	5	American Staffordshire Terrier	M	Negative	Negative	–	–	Non-infected	6.6	No

IFA, indirect immunofluorescent assay; UPC, urine protein:creatinine ratio; RIN, RNA integrity number. Samples with a RIN > 7 were included in the RNA-seq.

### Sample Distribution Based on Principal Component Analysis

After mapping to canine transcriptome, relationships between samples were evaluated by PCA, which explained 63% and 10% sample variability (the first and second principal component, respectively). The distribution of the samples was divided into two clusters and no outlier samples were detected (**Figures 1B, C**).

### Proportions of Immune Cell Subpopulations in Lymph Nodes

**Figure 2:**
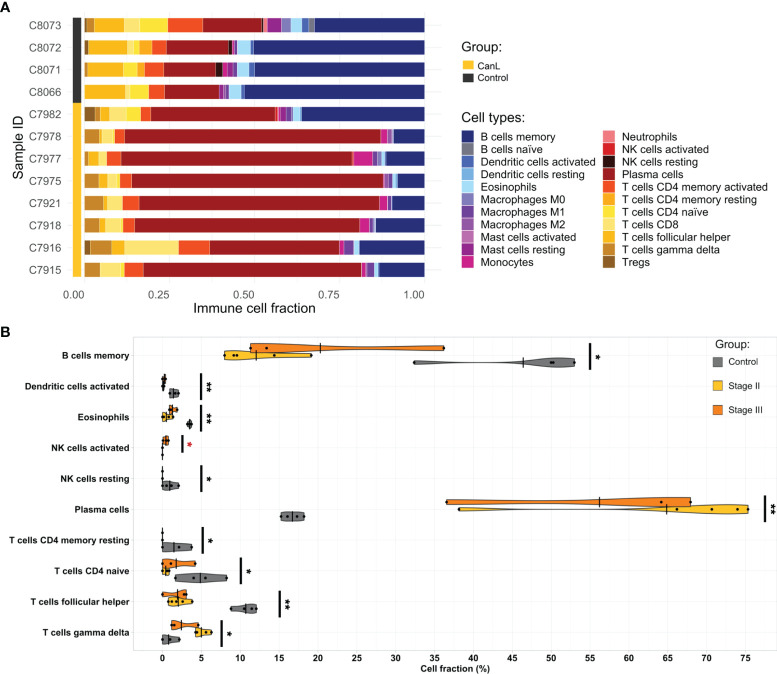
The landscape of the cell composition in the lymph node aspirates based on their RNA-seq data. **(A)** Relative proportion of each immune cell type across samples was inferred by CIBERSORTx. **(B)** Violin and dot plots of immune cell populations from deconvolution analysis that displayed significant differences between groups, as estimated by Wilcoxon test. Significant differences between clinical stages II and III are highlighted in red. *p-value <0.05, **p-value <0.01, ***p-value <0.001.

All samples were significant (p-value < 0.05) at the deconvolution for immune cell subset identification and considered acceptable for further analysis. CanL samples had higher proportions of plasma cells, CD8+ T cells, γδ T cells, monocytes and M1 macrophages, and lower levels of memory B cells, resting memory CD4+ T cells, naïve CD4+ T cells, follicular T helper cells, activated dendritic cells, resting NK cells and eosinophils than control samples. In addition, lymph nodes of dogs with CanL in stage III showed higher amounts of activated NK cells and tended to present higher proportions of M0 macrophages (**Figure 2**).

### Differentially Expressed Genes in Lymph Nodes

A total of 20,772 genes were expressed in lymph nodes, of which 14,134 had a normalized counts across samples ≥ 5 (**Table S2** and **Figure 1D**). Comparison of gene expression between case and control samples revealed 5,461 significantly differentially expressed genes (**Table S2**); 2,689 had a positive log_2_ fold change (log_2_FC) and 2,772 had a negative log_2_FC. In addition, 132 of the 20,772 genes were lncRNAs, of which 21 were significantly differentially expressed.

### Enriched GO Terms in the Differentially Expressed Genes

To understand the functional role of relevant genes during CanL, gene ontology (GO) enrichment analysis were performed to explore the biological functions of the 2,689 DEGs with a positive log_2_FC and the 2,772 DEGs with a negative log_2_FC. Complete results of functional enrichment analyses are provided in (**Table S3**).

**Figure 3:**
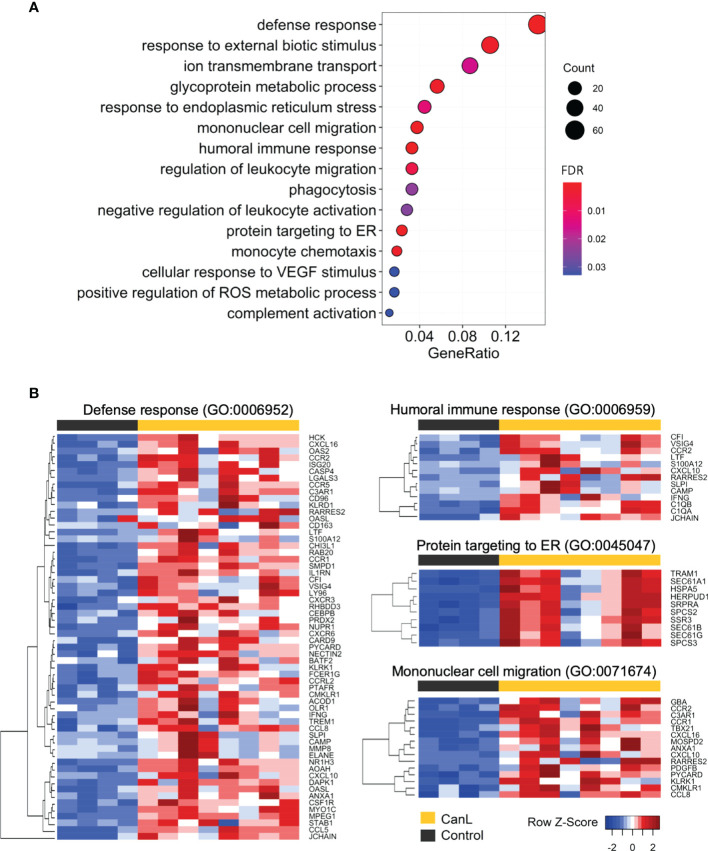
GO enrichment of the differentially expressed genes with positive log_2_FC. **(A)** All enriched GO categories of biological process in the differentially expressed genes with positive log_2_FC are displayed. The significance levels (FDR p-value) are represented by the color saturation, the size of the dots represents the number of genes in the gene set associated with the GO term and the gene ratio is represented by the horizontal bar length. **(B)** Gene expression heatmaps of the four most significant GO terms enriched in the differentially expressed genes with positive log_2_FC. Red through blue color indicates high to low expression.

**Figure 4:**
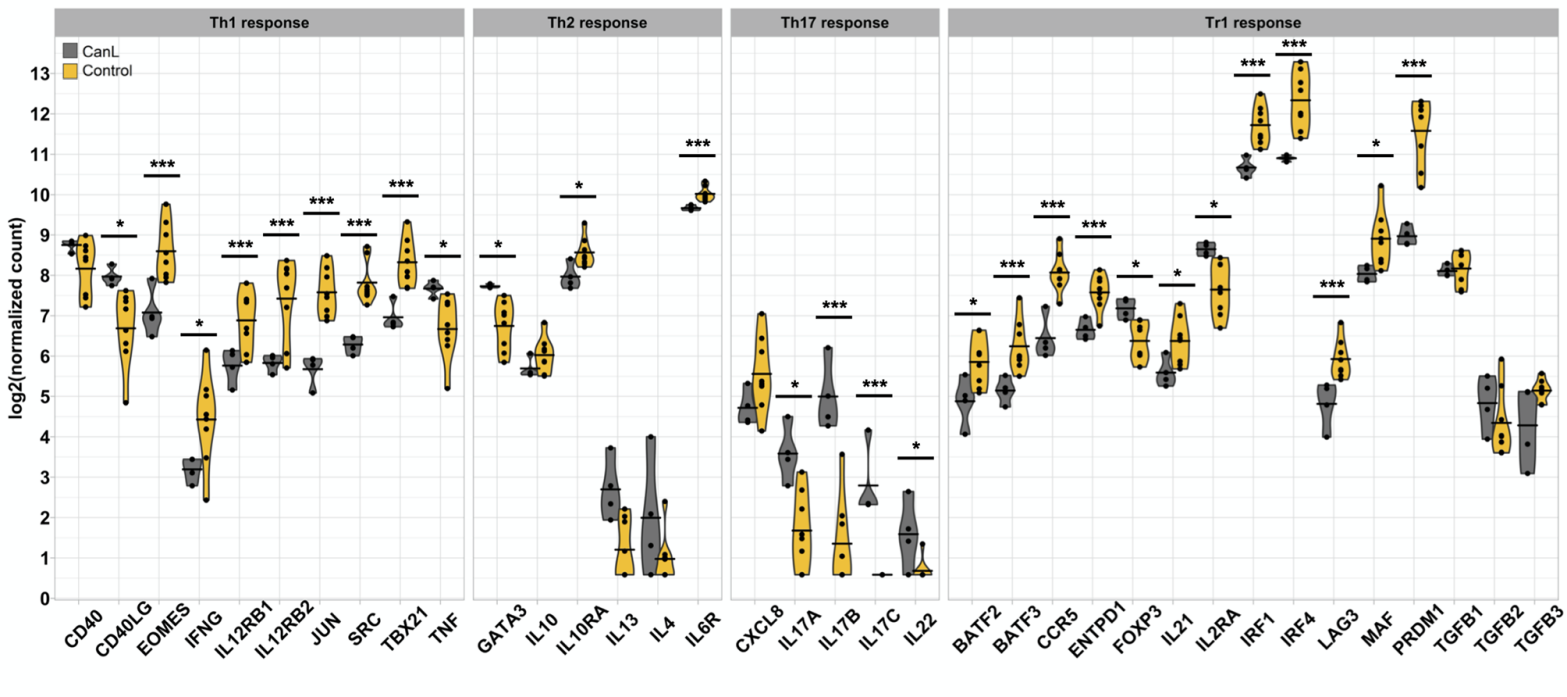
Th1, Th2, Th17, Tr1 gene expression signatures observed in the lymph nodes. Violin and dot plots of selected genes involved in Th1, Th2, Th17 and Tr1 immune responses during canine leishmaniosis. *p-value <0.05, **p-value <0.01, ***p-value <0.001.

In the DEGs with higher expression in CanL group, the enriched GO terms in biological processes were related to defense and immune responses (**Figures 3**, **4** and **Table S3**), including: humoral immune response, mononuclear cell migration, myeloid leukocyte activation and phagocytosis, among others; moreover they are also associated with response to endoplasmic reticulum (ER) stress and unfolded protein response (UPR), mediated by the activation of ER stress sensor inositol-requiring transmembrane kinase/endonuclease 1 (IRE1).

In the DEGs with lower expression in CanL group, cell-cell adhesion *via* plasma membrane adhesion molecules, transmembrane transport and cell-cell signaling, including trans-synaptic signaling, were the most enriched GO terms in biological processes (**Table S3**).

### Gene Co-Expression Modules of Lymph Nodes Correlate With Clinical Traits

**Figure 5:**
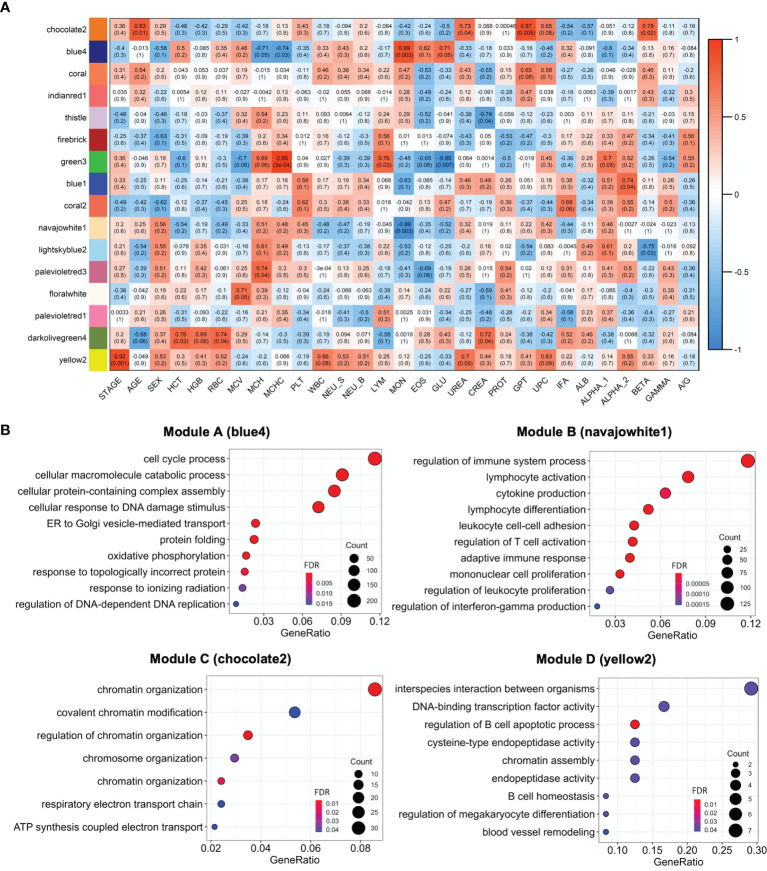
**(A)** Heatmap of the correlation of WGCNA modules with clinical traits. Red through blue color indicates positive to negative correlation. HCT, hematocrit; HGB, hemoglobin; RBC, red blood cells; MCV, mean corpuscular volume; MCH, mean corpuscular hemoglobin; MCHC, mean corpuscular hemoglobin concentration; PLT, platelets; WBC, white blood cells; NEU_S, segmented neutrophils; NEU_B, band neutrophils; LYM, lymphocytes; MON, monocytes; EOS, eosinophils; GLU, glucose; CREA, creatinine; PT, total protein; GPT, glutamate pyruvate transaminase enzyme; UPC, urine protein,creatinine ratio; IFA, indirect immunofluorescent assay; ALB, albumin; ALPHA_1, ALPHA_2, BETA, GAMMA, serum globulin fractions; A/G, albumin,globulin ratio. **(B)** GO enrichment of the A, B, C and D modules. The top 10 enriched GO categories of biological process were selected with clusterProfiler package to avoid redundant GO terms (showCategory = 10 and simplify = 0.5, 0.65, 0.95 and 0.75 for A (blue4), B (navajowhite1), C (chocolate2) and D (yellow2) modules, respectively). The significance levels (FDR p-value) are represented by the color saturation, the size of the dots represents the number of genes in the gene set associated with the GO term and the gene ratio is represented by the horizontal bar length.

Weighted gene co-expression network analysis was performed on 12,155 genes, using a soft threshold power (β) of 12 (scale free R^2^ = 0.85) to ensure a scale-free network (**Figure S1**). Initially, 187 modules were partitioned by dynamic tree cutting. Then, the eigengenes of each module (ME) were identified and merged into 16 main modules by applying a merging distance threshold of 0.45, which is a strict cut-off value that supported the reliability of the module divisions (**Figure S2**). Finally, 4 of these modules containing 6,835 expressed genes were associated with clinical traits related to the infection (correlation > 0.6 and a p-value ≤ 0.01) (**Figure 5A** and **Table 2**), and were considered key modules associated with CanL and worthy of further exploration, including: A module containing 3,706 genes; B module containing 2,302 genes; C module containing 779 genes and D module containing 48 genes (**Table 2**). Module eigengenes of A, C and D modules correlated positively with blood monocyte concentration, GPT levels and clinical stage, respectively, while B module correlated inversely with blood monocyte concentration (**Figure 5A**).

**Table 2:** Summary information of the key co-expression modules detected in the lymph nodes of dogs suffering from CanL.

Module ID	Color	Genes	Biological process	Clinical trait (correlation)
A	blue4	3,706	Cell cycle, ER stress and UPR	Blood monocyte concentration (0.89)
B	navajowhite1	2,302	Regulation of immune response	Blood monocyte concentration (-0.89)
C	chocolate2	779	Chromatin organization	GPT levels (0.87)
D	yellow2	48	Regulation of B cell apoptosis	Clinical stage (0.92)

### Enriched GO Terms in the Gene Co-Expression Modules

The key modules were characterized based on the most significant GO categories for biological processes (**Figure 5B** and **Table S3**). Thus, the A module was associated with cell cycle process, ER stress and UPR; the B module with the regulation of immune response, leukocyte differentiation, activation and adhesion; the C module with chromatin organization; and the D module with the regulation of B cell apoptotic process (**Table 2**).

## Discussion

In this study, we integrated whole transcriptome profiling and bioinformatics analysis for identifying regulatory pathways in canine lymph nodes associated with *L. infantum* natural infection. This approach allowed us to get a deeper understanding of the molecular mechanisms underlying the immunopathogenesis of CanL and identified four key modules associated with the disease, as well as several candidate genes which may contribute to CanL. To the best of our knowledge, this is the first study in the dog that evaluates the impact of *L. infantum* natural infection on the immune response at whole transcriptome level.

### Induction of Endoplasmic Reticulum Stress and Unfolded Protein Response

The ER is an organelle responsible for the synthesis, folding and modification of proteins (51). However, the capacity of ER functions can be exceeded under certain circumstances, such as infections by intracellular parasites (52, 53), and lead to ER stress due to the accumulation of misfolded proteins (54, 55). This triggers the UPR, one of the main protective mechanisms of the ER to resolve stress and dysfunction, which is also necessary for the physiological function of the innate immune system (56).

In this study, ER stress and UPR signaling, specifically the IRE1 and ER-associated degradation (ERAD) pathways, were found: a) associated with CanL samples (**Figure 3**); b) linked to module A from the WGCNA analysis; c) correlated with higher blood monocyte concentrations in sick dogs (**Figure 5** and **Table 2**); d) which also tended to correlate with increased monocyte abundances by cell deconvolution analysis (p-value = 0.074); and most important e) with increased M0 macrophage proportions in dogs in stage III of disease (p-value = 0.067) (**Figure 2**). The IRE1 branch of the ER stress response is a key signaling pathway in modulating innate and adaptive immune responses (56, 57). It is highly expressed in macrophages, T cells, plasma cells, dendritic cells, and NK cells in response to external stimuli, and regulates the production of proinflammatory cytokines through the activation of the X-box binding protein 1 (XBP1) (57–59). Thus, we could hypothesize that dysregulation of UPR may contribute to the inflammatory/regulatory imbalance during clinical leishmaniosis.

The role of ER stress in macrophages infected by *L. infantum* was evaluated by Galluzzi et al. (60), who showed a significant up-regulation of *XBP1*, suggesting that the parasite could promote survival of host cells by inducing a mild ER stress response. Similarly, we confirmed for the first time in naturally infected dogs a significant induction of ER stress and UPR, with an increased expression of *XBP1*. Other genes with a significantly higher expression involved in the IRE1 branch were *ERN1*, *EDEM1*, *DNAJB9*, *WIPI1*, *HYOU1* and *PDIA5*. Therefore, IRE1 could be a critical pathway implicated in the immunopathogenesis of CanL.

*Vascular endothelial growth factor-A* (*VEGFA*) had also a significantly positive log_2_FC. Endothelial growth factor family members induce changes in the vascular network during inflammation. In fact, VEGFA can mediate inflammation-induced lymphangiogenesis and have intense effects on lymph nodes (61–65). IRE1 powerfully regulates *VEGFA* expression under various stress conditions (66–68), and infected macrophages harboring *Leishmania donovani* release extracellular vesicles that induce endothelial cells to secrete VEGFA (69). Weinkopff et al. (70) also found that infection with *Leishmania major* increases the expression of *VEGFA* and *VEGF receptor-2* (*VEGFR-2*) at the site of infection, and it correlated with lesion size and parasite burden, suggesting that VEGFA-dependent lymphangiogenesis could be a mechanism that restricts tissue inflammation and contributes to the severity of leishmaniosis.

### Modulation of Th1 and Th2 Expression Profiles

Defense response is the most relevant biological process modified in the lymph nodes of dogs with CanL, and is highly enriched in the DEGs with positive log_2_FC (**Figure 3**), with six of the ten higher expressed DEGs (*CHI3L1, SLPI, ACOD1, CCL5, MPO* and *BPI*) involved in immune responses against pathogens (71–76). Moreover, we observed a significantly decreased expression of *tumor necrosis factor-α* (*TNF-α*) (**Figure 4**). This cytokine is an essential component of the Th1 response, as it contributes to trigger nitric oxide production in activated macrophages, and therefore, its deficit may increase the severity of leishmaniosis (77). However, a significant increase in the expression of *IFN-γ*, the canonical Th1 cytokine, was found in the CanL group (**Figure 4**). The expression of some key factors for IFN-γ production were significantly increased too, including *T-box expressed in T cells* (*TBX21*), *eomesodermin* (*EOMES*), *AP-1 transcription factor subunit* (*JUN*), *Src kinase* (*SRC*), *β1 and β2 subunits of the IL12 receptor* (*IL12RB1*; *IL12RB2*) (**Figure 4**). Nevertheless, even with high IFN-γ levels, the host may fail to control the infection, probably due to an incomplete response to IFN-γ (78).

Although the expression of *IL-10* was slightly elevated, the *IL-6 receptor* (*IL6R*) and the *subunit α of the IL-10 receptor* (*IL10RA*) were the only Th2 interleukin-related molecules significantly increased in the CanL group. In contrast, *IL-4* and *IL-13* showed a tendency to decrease in infected dogs (**Figure 4**). Alves et al. (79) detected a significant increase of *TGF-β* and *IL-10* expression in the lymph nodes of dogs with CanL, but their expression remained stable in our study, with dogs in stage III displaying just a slightly higher expression of *IL-10* than those in stage II. The expression of Th2 cytokines probably increase in very severe CanL (clinical stage IV). Thus, it could not be detected here, as we only included dogs with moderate to severe disease (clinical stages II-III). In fact, we found the expression of *GATA3* significantly decreased in the CanL group (**Figure 4**). Among other functions, this transcription factor is critical for the induction of Th2 cytokine production and growth of Th2 cells (80), supporting a lack of Th2 response and a less relevant role of Th2 cells in the lymph nodes at these stages of the infection. However, this hypothesis could not be confirmed, since the clinical staging was not performed by Alves et al. (79), and the transcriptional changes we observed may not directly correlate with cell functionality.

Along with the dysregulated expression of pro- and anti-inflammatory interleukins, significant changes in the expression of some chemokines and chemokine receptors were also detected: *CCL3*, *CCL4, CCL5, CCL8, CCL22, CXCL10*, *CCR1, CCR2* and *CCR5*. These chemokines play a vital role in determining the Th1/Th2-mediated responses (81), and can represent a potential prophylactic and therapeutic target to promote immune clearance of the parasite in CanL, specially *CCL5*, as it is one of the ten most expressed DEGs.

### Suppression of Th17 Response and Neutrophil Activation

Th17 cells are an additional type of CD4+ T helper cells contributing to defense response, mainly through the production of IL-17. This interleukin synergizes with CCL3 and acts as a potent activator of neutrophils (82–85). Therefore, the continual production of IL-17 during clinical CanL may lead to an over-recruitment of neutrophils to inflammatory sites (86), which could result in the slightly higher expression of *IL-8* we observed. The overexpression of *IL-8* may promote parasite persistence, as this cytokine induces a massive and long-lasting accumulation of neutrophils (87, 88), where the parasite may survive (12, 22, 23, 89). However, in an experimental model of canine *L. infantum* infection, IL-17 transcription was reduced in lymph nodes, suggesting that the hyperinflammatory response generated at the beginning of the infection was suppressed as the disease progressed (90). This is also in accordance with our results, since three related members of the IL-17 gene family (*IL17A, IL17B* and *IL17C*), as well as *IL-22*, had a significantly lower expression in the case group (**Figure 4**).

### Activation of Tr1 Response

The peripherally derived regulatory T cell subset CD4^+^ CD25^-^ Foxp3^-^ type 1 regulatory T (Tr1) cells are induced by chronic activation of CD4+ T cells by antigens in the presence of tolerogenic conditions (91–93). These Tr1 cells suppress the host immunity and down-regulate the activation and proliferation of effector T cells, including Th1 (12, 92–94). Increased activation of IL-10 producing Tr1 cells has been shown in chronic cutaneous leishmaniosis in humans, as well as in murine visceral leishmaniosis (95–98), although no changes in the expression of *IL-10* and *TGF-β* were detected in CanL (99).

Interestingly, we observed a significantly lower expression of CD25 (*IL2RA*) and *FOXP3* (**Figure 4**), which could be consistent with an increase in the number of Tr1 cells in the lymph nodes of CanL dogs, with no variation in the expression of *IL-10* and *TGF-β*. Furthermore, the expression of several Tr1 markers and transcription factors were also significantly higher (*LAG3, IRF1, IRF4, CCR5, BATF, MAF, PRDM1, ENTPD1* and *IL21*) (**Figure 4**). Many of these genes were included within the B module, which negatively correlated with the concentration of circulating blood monocytes and was enriched in GO terms related to lymphocyte activation and proliferation (**Figure 5**). As it was previously described, *L. infantum* may induce the activation of Tr1 cells to suppress the inflammatory response of the host (91, 93, 100), and we hypothesize that this suppression could potentially reduce the abundance of the blood monocyte population, although further research is needed to confirm it.

### Exhaustion of T and NK Cells

T cell exhaustion is a state of dysfunction triggered by a prolonged antigen exposure during many chronic infections that prevents optimal control of pathogens, including intracellular parasites (101–105). Indeed, T cell exhaustion has already been described in CanL, where T cell proliferation and functionality decreased as disease progresses (106). CD8+ T cells typically show an impaired cytotoxic activity, and lose the ability to produce IL-2 and TNF during the first stages of exhaustion, while severe exhaustion may lead to a completely lack of the ability to produce IFN-γ, CCL chemokines or to degranulate. More severe CD8+ exhaustion correlates with higher antigen load and loss of help from CD4+ T cells (105, 107). Here, we observed a lower expression of *TNF* and *IL-2 receptor*, as well as a higher expression of several transcriptional markers previously associated with the T cell exhaustion process, such as: surface inhibitory receptors and their ligands (*LAG3, CD244, CD160, Fas* and *Fas ligand, TRAIL*, and four TNF receptors), *IL-10 receptor*, and some downstream transcription factors (*Blimp-1, EOMES, BATF* and *JUN*) (102, 105), together with the Tr1 transcriptional markers mentioned above. In addition, *LAG3, TRAIL* and *Blimp-1* (*PRDM1*) were also detected in the A co-expression module, which was significantly associated with cell cycle processes. However, these transcriptional factors can be expressed by other cell types and could exert additional functions.

On the other hand, we found that the expression of *IL-15* was slightly but significantly increased in the case group. In lymph nodes, this cytokine is produced by APCs and it promotes the development and function of NK cells, priming them for cytolytic activity and production of IFN-γ (108–110). In fact, we found that the proportion of activated NK cells was significantly higher in the lymph nodes of dogs with CanL in stage III than in stage II (**Figure 2**). Although we noted overexpression of genes related to cytotoxic activity of NK cells, including *Fas* (*FAS*), *granzyme A* (*GZMA*)*, granzyme B* (*GZMB*), *perforin 1* (*PRF1*), *natural killer receptor 2B4* (*CD244*) and *Killer Cell Lectin Like Receptor K1* (*KLRK1*), the expression of *CD69*, a marker of NK cell activation (111, 112), was lower in CanL dogs, compared with the control group.

These results are compatible with an exhausted phenotype or bystander activation of CTLs and NK cells during CanL, which could lead to incomplete activation of these cells, linked with lower production of Th1 cytokines and enhanced cytotoxic molecule expression. Similar changes are frequently observed in chronic infections involving high levels of persistent antigen (94, 107). In fact, a strong induction of cytotoxic transcriptional signature associated with CTL and NK cell senescence was found in cutaneous leishmaniasis lesions (113). This cytolytic transcriptional signature correlated with treatment outcome (114), and it was also found in the blood of *L. braziliensis* patients (115), suggesting that dysfunctional states of T and NK cells may have a major role in the immunopathology of *Leishmania* infections.

### Impaired Activation and Dysfunctions of Monocytes and Macrophages

Monocytes and macrophages are the final host cells for *Leishmania* and, therefore, these cells are crucial to disease progression (116, 117). Here, we found that the B module was inversely correlated with the concentration of circulating blood monocytes (**Figure 5A** and **Table 2**), which could be partially explained by the fact that it was functionally enriched in leukocyte adhesion GO terms, as shown in **Figure 5B**. A high number of DEGs with positive log_2_FC were also involved in mononuclear cell migration, specifically in monocyte chemotaxis (**Figure 3**), such as *AIF1*, *CCL3*, *CCL4*, *CCR2*, *ICAM1*, *ICAM2, RAP1A, ITGB2, SELPG* (118). We hypothesis that the increased expression of these genes in lymph nodes may promote the extravasation of monocytes from blood vessels and their migration into infected tissues, where their interaction with the parasite could lead to their activation into functional macrophages (12).

Although IFN-γ is critical for this activation and control of macrophage infection by *Leishmania* species, mainly by inducing the release of NO, the complete activation of macrophages to effector cells requires CD40-CD40 ligand (CD40LG) interactions (12, 119). However, the expressions of *CD40* and *CD40LG* were both decreased in the CanL group, although only CD40L (*CD40LG*) reached a statistically significant value (**Figure 4**). For instance, *CD40LG*, *TNF-α* and *IFN-γ* were all detected in the B co-expression module, which was associated with the regulation of immune response and correlated with blood monocyte concentration, suggesting that the expression of these genes may influence the dynamics of monocytes and macrophages during *L. infantum* infection.

In addition to CD40-CD40L, the activation of mitogen-activated protein kinases (MAPKs) is needed to induce the production of proinflammatory cytokines and NO in macrophages (120–122). Interestingly, the disruption of the MAPK signaling pathway is frequently observed during *Leishmania* infections (6, 123–125) and we found *MAPK4* among the ten DEGs with the lowest expression.

Another potential mechanism contributing to dampen specific immune activation during *L infantum* infection in the lymph nodes of dogs suffering from CanL may be the higher expression of *serine leucocyte proteinase inhibitor* (*SLPI*) they displayed, as it is a potent myeloid-derived anti-inflammatory and microbicidal molecule that targets monocytes and macrophages to modulate innate and adaptive immune responses (126–128), and has been shown to dysregulate the M1/M2 response during other *Leishmania* infections (129).

Moreover, we observed a higher expression of the *IL-1 receptor antagonist* and a lower expression of the *Nod-like receptor protein 3* in the CanL group. These genes has been previously shown to contribute to the suppression of inflammatory responses and NO production *via* signaling through IL-1R, favoring the parasite survival in macrophages (130–132). However, additional mechanisms affecting these or other molecules cannot be ruled out, such as receptor instabilities, post-translational modifications, diminished DNA binding activity of transcription factors or the influence of *Leishmania* exosomes on immune cells, among others (133, 6, 134).

Overall, our results may suggest that the upstream signaling events leading to the production of IFN-γ are expressed in the lymph nodes of dogs with CanL, but we did not find any significant variations in the expression of *inducible nitric oxide synthase* (*iNOS*) or *arginase 1* (*Arg1*), the two main enzymes involved in NO metabolism during the infection of macrophages by *Leishmania* spp. (5, 135, 136).

On the other hand, it is worth noting that *solute carrier family 11 member 1* (*SLC11A1*), formerly known as *natural resistance-associated macrophage protein 1* (*NRAMP1*), was identified among the overexpressed DEGs involved phagosome maturation (137). This gene has been widely investigated for its potential role in susceptibility to leishmaniosis (138), as it pumps the metal ions out of the parasitophorous vacuole (139) to deprive the parasite of iron and block its development (140).

### B Cell Dysfunction and Humoral Immunity

Impaired humoral immunity could play a critical role in the progression of CanL, as hypergammaglobulinemia, nonspecific polyclonal antibodies and circulating immune complexes correlate with clinical progression of the disease (12, 141–143). However, participation of B cells in the immunopathogenesis of leishmaniosis is not only related to antibody production, but also to their functions as regulatory and APCs (144).

Human visceral leishmaniosis is associated with an increased expression of *Blimp-1*, which dampen the antigen presentation machinery in B cells and promotes their differentiation into plasma cells, leading to the observed hypergammaglobulinemia during clinical disease (143). The overexpression of two anti-apoptotic and survival signals for plasma cells are also key in the immunopathogenesis of visceral leishmaniosis: *B-cell maturation antigen* (*BCMA*) and *transmembrane activator, calcium modulator and cyclophilin ligand interactor* (*TACI*) (143, 145, 146). Our results were in line with these findings, as the three markers (*Blimp-1, BCMA* and *TACI*) were highly expressed in the CanL group, and hypergammaglobulinemia was observed in these patients (**Table S1**), which also presented higher proportions of plasma cells (**Figure 2**). Furthermore, we detected a co-expression module, the D module, associated with the regulation of B cell apoptotic process and significantly correlated with the clinical stage of CanL (**Figure 5** and **Table 2**), suggesting that B cell homeostasis could be a key factor in the progression of CanL. All these provided clues regarding the involvement of B cells in promoting leishmaniosis, as they may have compromised abilities and would produce high antibody titers, which are detrimental during the chronic infection.

### Regulation of Gene Transcription: LncRNAs and Chromatin Organization

Finally, it is worth noting that lncRNAs might participate in the immunopathogenesis of CanL, as they are critical regulators of gene transcription during immune response through regulating protein-protein interactions or *via* their ability to assemble with RNA and DNA (147–150). Accordingly, we found that changes in the expression of several lncRNAs were significantly associated with CanL, including an *antimicrobial peptide NK-lysin-like* (*LOC608395*) and *JUN*, which play immunomodulatory roles (151, 152). In particular, *JUN* could be a key immune regulator during *L. infantum* infection, since it is the second most differentially expressed lncRNA in the lymph nodes of dogs with CanL and regulates proinflammatory cytokines, chemokines and NO production (152), essential to achieve parasite control.

Chromatin organization is also crucial for transcriptional regulation in the immune system (153, 154). In fact, the immune response induced by antigen exposure led to an increase in the level of chromatin activation and RNA content in the popliteal lymph nodes of dogs (155), suggesting that modification in the chromatin structure is essential to mount an effective immune response. Therefore, regulation of chromatin organization could be targeted by intracellular parasites to evade their host defense mechanisms. For instance, some parasites provoke changes in the chromatin states through sequence-specific DNA-binding proteins or ncRNAs to inhibit inflammatory responses (156). Here, we identified a significant association between the C co-expression module and the chromatin organization GO term (**Figure 5B** and **Table 2**). This module was also correlated with age and glutamate-pyruvate transaminase (GPT) serum levels (**Figure 5A**), which is not surprising, as aging influences both chromatin structure (157) and liver function (158). Then, changes in chromatin organization are likely to contribute to shaping the immune response during *Leishmania* infection, and age could impact this immune modulation, although the precise underlaying mechanisms involved in the pathogenesis cannot be established at this stage of the study.

## Conclusion

In summary, we identified 5,461 differentially expressed genes and four key modules involved in several biological processes related to immune responses in dogs with CanL. Altogether, these data showed that *L. infantum* infection induces strong transcriptional changes in canine lymph nodes. These alterations could regulate host immunity at multiple levels to promote parasite persistence, such as: increasing host cell survival through the expression of the IRE1 branch of the UPR; dysregulating the expression of cytokines which are key in determining Th1, Th2, Th17 and Tr1-mediated responses; fostering T cell and NK cell exhaustion processes; and disrupting monocyte, macrophage and B cell activation and functionality. Furthermore, *L. infantum* infection could influence gene transcription by modulating lncRNA’s expression profiles and chromatin organization. Further investigation into these biological processes may lead to new immunomodulatory strategies to control *Leishmania* infections. Future studies are also warranted to further characterize the role of differentially expressed lncRNAs in the immunopathogenesis of CanL, as they display the potential to be targets for immunotherapy.

## Data Availability Statement

The datasets presented in this study can be found in online repositories. The names of the repository/repositories and accession number(s) can be found below: https://www.ebi.ac.uk/ena, PRJEB47771.

## Ethics Statement

The animal study was reviewed and approved by the Experimentation and Animal Welfare Committee of the Complutense University. Written informed consent was obtained from the owners for the participation of their animals in this study.

## Author Contributions

CS, AR-P, NS, SD and GM conceived the project. CS, NS, and SD acquired funding. CS and GM acquired samples and clinical information of the patients. CS conducted the experiment. CS and AR-P performed the bioinformatic analyses, data visualization and wrote the original draft. All authors reviewed and approved the final version of the manuscript.

## Funding

CS acknowledges the financial support for her Predoctoral Fellowship for Research Personnel in Training UCM of the Universidad Complutense de Madrid (Spain) and Banco Santander (CT42/18-CT43/18). AR-P has been supported by the Research Program “Atracción de Talento de la Comunidad de Madrid” (2017-T2/BMD-5532), and granted by the Regional Programme of Research and Technological Innovation for Young Doctors UCM-CAM (PR65/19- 22460).

## Conflict of Interest

The authors declare that the research was conducted in the absence of any commercial or financial relationships that could be construed as a potential conflict of interest.

## Publisher’s Note

All claims expressed in this article are solely those of the authors and do not necessarily represent those of their affiliated organizations, or those of the publisher, the editors and the reviewers. Any product that may be evaluated in this article, or claim that may be made by its manufacturer, is not guaranteed or endorsed by the publisher.
