# Supplementary figures and images for: Modulation of Host Immune Response during Leishmania infantum Natural Infection: A Whole-Transcriptome Analysis of the Popliteal Lymph Nodes in Dogs
Source: Front Immunol. 2022 Jan 4;12:794627. doi: 10.3389/fimmu.2021.794627 (PMC8763708; doi:10.3389/fimmu.2021.794627)

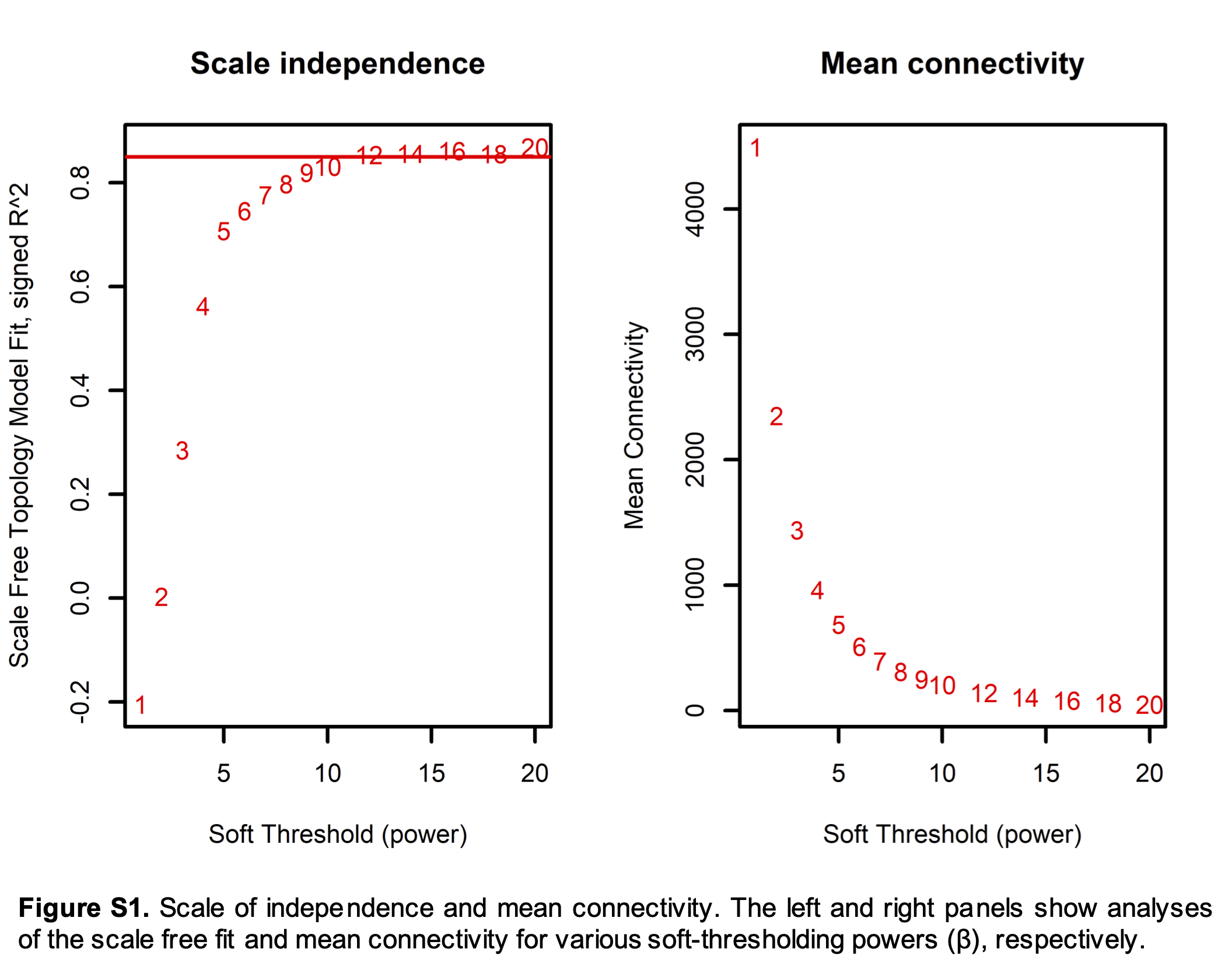

Supplement: Supplementary file 1 [file Image_1.tiff]

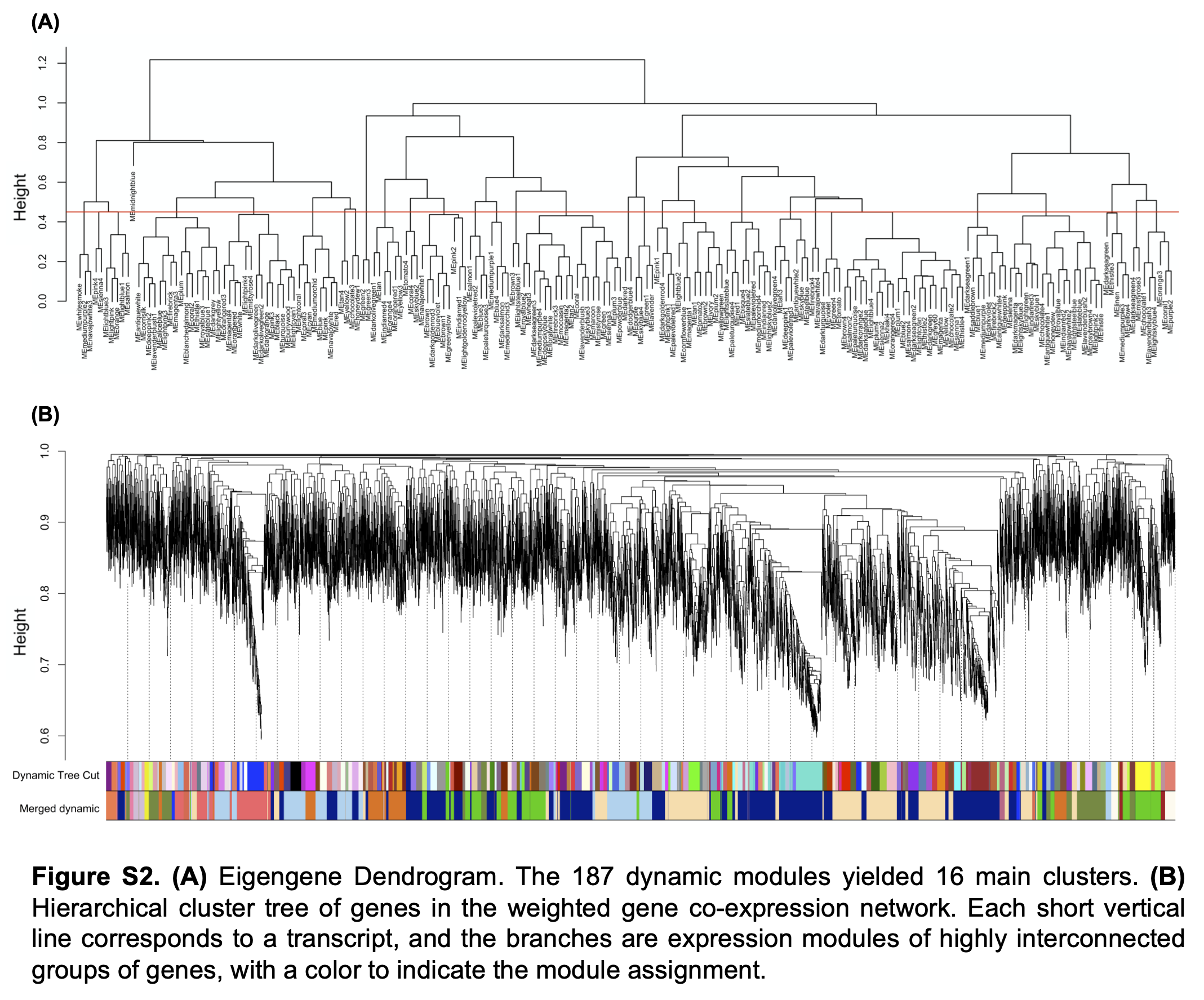

Supplement: Supplementary file 2 [file Image_2.tiff]
